# Willingness for video visits for non-emergent health issues, mental health, or specialty visits

**DOI:** 10.3389/fdgth.2026.1845428

**Published:** 2026-08-27

**Authors:** Paige Carlson, James Merchant, Julia Friberg Walhof, Kelly Miell, Peter J. Kaboli, Stephanie L. Shimada, Jasmine Mares, Amy M. J. O’Shea

**Affiliations:** 1 Department of Internal Medicine, University of Iowa Carver College of Medicine, Iowa City, IA, United States; 2 Department of Biostatistics, University of Iowa College of Public Health, Iowa City, IA, United States; 3 Center for Access and Delivery Research and Evaluation (CADRE), Iowa City VA Healthcare System, Iowa City, IA, United States; 4 VHA Office of Rural Health, Veterans Rural Health Resource Center-Iowa City (VRHRC-IC), Iowa City Veterans Affairs Healthcare System, Iowa City, IA, United States; 5 Center for Healthcare Organization and Implementation Research (CHOIR) at the Bedford VA Medical Center, Bedford, MA, United States; 6 Department of Health Law, Policy and Management, Boston University School of Public Health, Boston, MA, United States; 7 Division of Health Informatics and Implementation Science, Department of Population and Quantitative Health Sciences, University of Massachusetts Chan Medical School, Worcester, MA, United States

**Keywords:** healthcare delivery, internet, survey, telemedicine, veteran

## Abstract

**Importance:**

While there is growing evidence for the benefits of, and individual facilitators to, telemedicine use, few studies evaluate factors influencing willingness to engage with video telemedicine.

**Objective:**

To assess factors associated with willingness to participate in video telemedicine for non-emergent health issues in outpatient settings.

**Design:**

Survey study using quantitative and qualitative data collected through categorical and open-ended questions via a computer-assisted telephone interview conducted from April to October 2022.

**Setting:**

The Veterans Health Administration (VHA)

**Participants:**

350 veterans with a VHA primary care visit in March 2022

**Exposures:**

The survey asked veterans about their internet access, phone, and internet-capable devices, overall telemedicine (i.e., medical visits by telephone or video) experience and preferences. Demographics, financial resources, living situation, transportation, and basic needs were also reported.

**Main outcomes:**

The primary outcome was self-reported willingness to participate in an outpatient video visit with a doctor for a non-emergent: health issue, mental health outpatient visit, or health issue requiring a specialty physician. Outcomes were assessed using sample weighted ordinal logistic regression and thematic analysis for the open-ended questions.

**Results:**

Overall, 264 (76.1%) of survey respondents were willing to use video telemedicine for a non-emergent health issue. In mental health or specialty care settings, respectively, 241 (70.7%), and 212 (61.6%) of survey respondents were willing to use video telemedicine. Though many Veterans reported telemedicine benefits (i.e., convenience, reduced travel burden, easy medication renewal), others were reluctant or unable to use the technology (i.e., digital literacy, insufficient internet). In adjusted analyses, willingness to participate in video telemedicine was associated with a differentially higher probability of expressing interest in telemedicine but not knowing how to use it in each setting [health care issue: 12.0%; 95% CI = (1.3%, 22.8%); mental health: 16.6%; 95% CI = (5.3%, 28.0%); specialty care: 21.1%; 95% CI = (5.9%, 36.4%)].

**Conclusions and relevance:**

While willingness to choose video telemedicine is influenced by multiple factors and was dependent on the setting, knowing how to use the technology was a barrier in all three clinical settings for those interested in telemedicine. Training opportunities to improve know-how with video telemedicine technologies should be considered.

## Introduction

Minimizing in-person care ensured patient and provider safety during the Covid-19 pandemic. Consequently, healthcare systems adapted to providing patient care via alternative means; typically, via telemedicine (i.e., video or telephone) (1, 2). With extensive telemedicine experience (3, 4), the Veterans Health Administration (VHA) rapidly offset in-person visits with video and telephone modalities (5, 6). Though VHA telemedicine use has decreased from its pandemic peak, telephone and video encounters across all services accounted for 36.7% of care in April 2023 (7). Importantly, Ferguson et al. also found video-based care, overall, remained relatively stable to peak levels, but varied meaningfully across clinic types [i.e., primary care, mental health (MH), and subspecialties]. And yet, specialized telemedicine programs have existed within VHA even prior to the pandemic and often address specific patient needs (e.g., clinical pharmacy (8), sleep care (9), hepatitis C treatment (10), ophthalmology services (11)) or system-level needs for underserved, typically rural, locations [e.g., contingency staffing programs (12), tele-ICU (13)]. Thus, willingness to participate in telemedicine may impact access to specific VHA services.

While there is growing evidence regarding the benefits (e.g., improved access to care, increased patient satisfaction, and reduced travel burden) (14–19), and individual facilitators to, telemedicine use (e.g., technical support, internet access, ease to use) (20, 21), literature regarding factors influencing willingness to engage with video telemedicine is limited. Most previous studies have focused on effectiveness, disparities in access, cost-effectiveness, and patient experience (22–29). Willingness to engage with video telemedicine depends on multiple factors. Perceived inadequacy of physical exam, decreased provider attention, barriers to asking questions, or a decreased ability to form a patient-provider relationship have been identified as concerns by patients and providers (30–33). Despite these concerns, studies show generally positive attitudes toward video telemedicine among patients and providers (34–37). The question then is how patients balance their concerns with video telemedicine while choosing the most appropriate way to receive their care.

Within the VHA, telemedicine has historically supported increased access to care for rural-residing veterans (38–40) or specific sub-specialties [e.g., cardiology (41), nephrology (17, 42), neurology (43)] with limited access in underserved areas. Despite the potential of such programs to mitigate higher geographic isolation, longer travel times, outside commitments, or unease in the VHA healthcare setting barriers including older age, digital literacy gaps, and limited internet or device access among veterans impede the realization of these benefits (5, 26, 44–46). Importantly, VHA has attempted to address these barriers thru investments in internet-enabled devices for qualifying veterans (47), and the implementation of Virtual Health Resource Centers (VHRC) which provide training for virtual care tools (48, 49). Even in this environment, the use of in-person, video, telephone, or some combination of these modalities varies by chronic condition (50) and patient preference (34, 51, 52).

This study, conducted among a population of rural and urban veterans, builds upon this work by assessing veterans’ willingness to use video telemedicine in three scenarios, i.e., for a non-emergent health issue, MH outpatient visit, or health issue requiring a specialty physician. This allows the assessment of extrinsic and intrinsic variables that may variably impact patient choices in different settings. Our hypotheses were patients will be willing to use video telemedicine for routine care, and outside factors, such as shorter time until appointment and comfort using technology, will encourage patients to prefer video over in-person care.

## Materials and methods

### Study design & participants

This mixed method secondary analysis was conducted using primary data collected from a computer-assisted telephone interview (53–55) conducted by the study team from April 25, 2022, through October 20, 2022.The study population was selected among veterans nationwide identified as having a VHA outpatient primary care visit, in-person or via VA Video Connect (VVC) in March 2022. Veterans were stratified equally by residential rurality (i.e., rural, urban) and primary care visit modality (i.e., in-person or VVC). Rural veterans and VVC visits were oversampled. Individuals with comprehension or hearing issues interfering with interview conduct were excluded. Participants were compensated $25 for interview completion with a $5 bonus if they called-in to schedule. Study recruitment concluded when *N* = 350 interviews were completed; 12.3% of veterans contacted (*N* = 2845).

Using the VA Informatics and Computing Infrastructure, primary care visits were identified using clinic stop codes and classified by modality. Residential rurality was defined by Rural-Urban Commuting Area Codes and dichotomized as rural (i.e., highly rural, rural, or isolated categories) or urban (56). Patient demographics including age, sex, race, Hispanic ethnicity, and comorbidity score (57) were also extracted. Comorbidity scores were calculated based on diagnoses documented from October 2021 through September 2022.

Survey weights were calculated to account for stratification, oversampling, and non-response using inverse probability of treatment weighting (51). We first calculated the inverse of the sample weights for each stratification group followed by a propensity score model among all veterans mailed a consent letter. This model adjusted for stratification (i.e., residential rurality and video visit use) and patient demographics (i.e., age, sex, race, Hispanic ethnicity, and comorbidity score). Respondents and non-respondents were compared by chi-square or t-test. Post-stratification weights were calculated by multiplying the propensity score weights by the design weights.

The study was approved by the University of Iowa Institutional Review Board and the VA Iowa City Research and Development Committee.

### Interview design and administration

The survey asked veterans about their access to the internet, phone, or other internet-capable devices, overall telemedicine (i.e., medical visits by telephone or video) experience and preferences. Questions about internet and device use were adapted from the Powhatan Broadband Survey (58) and the American Community Survey (59). Telemedicine engagement and satisfaction questions originated from a study by *Horrell* et al (60). Healthcare scenarios assessing situational preferences for in-person or video-based visits and veteran willingness to engage in a video visit were adapted from a previous study (61). Demographic information, financial resources, living situation, transportation, and basic needs were assessed via PRAPARE Survey (62). Survey questions were primarily dichotomous or multiple choice with each providing the option to respond “don't know” or “refused.” Open-ended questions are described within the qualitative analysis section below. The full computer-assisted telephone interview has been published elsewhere (51).

### Healthcare scenarios

Participants were presented with three healthcare scenarios in which they would see a doctor for a non-emergent health issue, MH outpatient visit, and health issue requiring a specialty physician (61). In each scenario, veterans were first asked if they would prefer an in-person or video visit if the appointment were offered on the same day. Second, they were asked their preference between an in-person visit a month from now versus a video visit next week.

### Primary outcomes

Participants rated their willingness to participate in a video visit with a doctor in each healthcare scenario (61). The five-point Likert scale was condensed into three categories, unwilling (i.e., “unwilling” and “slight unwilling”), “neutral”, and willing (i.e., “slightly willing” and “willing”).

### Quantitative analyses

Survey responses were stratified by willingness to have a video visit in each scenario. Continuous variables are reported with weighted domain means and 95% confidence intervals with a Taylor series (linearization) method to calculate the variance. Categorical variables are reported as unweighted counts with weighted percentages; differences were assessed using the Rao-Scott chi-square test, which corrects for survey weighting. An omnibus test of equality model coefficients within a regression framework assessed continuous variables.

We first considered ordinal logistic regression models for three domains: demographics and external factors, perceived ease of use, and perceived usefulness. Initially, the study team identified variables as potential determinants of patient willingness to participate in a video visit. All variables meeting an *a priori* 0.25 significance level threshold were included in the final modelling stage which used sample weighted ordinal logistic regression models. Average marginal effects and 95% confidence intervals are reported. A category of “non-response” was included for predictor variables with ≥5 non-responses (i.e., “don't know” or “refused”); else non-response was treated as missing (e.g., a complete case analysis was conducted).

The data analyses for this manuscript was generated using SAS software, Version 9.2 of the SAS System and STATA 18 software (63).

### Qualitative analyses

The interview asked open-ended questions about what made their telemedicine experience positive or negative (*N* = 284), why they felt their virtual medical visit was better, just as good, or worse compared to an in-person visit (*N* = 304), as well as whether they had any additional comments related to internet use, internet capable-devices, or telemedicine (*N* = 316). These responses were imported into a qualitative software platform (MAXQDA 2022) and assessed under the Technology Acceptance Model (TAM) framework as a guiding analytical lens (64). This model is widely used to predict user readiness to adopt new technology. In this model, external factors (e.g., system design features) trigger the user’s perception of technology’s usefulness and ease of use, which affects technology attitudes. The qualitative analyst (JFW) summarized key data points via inductive coding aligned with the TAM constructs, information categories were created, sorted into categories, and analyzed for patterns and themes (65). After initial coding, (JFW) created subcodes within parent codes containing ≥100 coded segments to identify additional themes within these larger categories. The codebook was reviewed with AMJO and SS to ensure consensus. Coded segments were exported, sorted by major themes, and summarized.

## Results

Overall, participants indicated they were willing to participate in a video visit for a non-emergent health issue (76.1%) (Table 1). In this scenario, those who were willing compared to unwilling were, on average, younger (Mean 60.0 vs 67.1 years), and more likely to indicate telemedicine visits would be easier with help getting access to the internet (25.5% vs. 20.7%) or a tablet (26.6% vs. 12.4%). Telemedicine visits by video or phone in the previous year were also more common among those who were willing versus unwilling to use video visits (84.9% vs. 20.2%) and they were more likely to report having a positive telemedicine experience (82.6% vs. 20.2%) or being comfortable with the technology (80.4% vs. 37.1%).

**Table 1:** Counts and weighted percentages for survey responses, stratified by veteran willingness to participate in a video visit for a Non-emergent health issue.

Survey Question	Unwilling *N* = 32	Neutral *N* = 51	Willing *N* = 264	p-value
**Demographics and External Factors, N (Weighted %)**				
Age^a^, Mean (95% CI)	67.1 (60.5, 73.7)	64.4 (60.1, 68.8)	60.0 (58.1, 61.8)	0.04
Comorbidity Score, Mean (95% CI)	0.6 (0.3, 0.9)	0.5 (0.3, 0.7)	0.6 (0.5, 0.7)	0.80
Residential Rurality				0.12
Urban	19 (72.0)	20 (54.0)	134 (68.4)	
Rural	13 (28.0)	31 (46.0)	130 (31.6)	
Female Gender	4 (9.0)	7 (10.4)	47 (13.5)	0.63
Race^a^				0.10
Black/African American	3 (14.3)	3 (9.8)	19 (9.6)	
White/Caucasian	23 (67.2)	47 (89.3)	208 (75.2)	
Other	2 (7.1)	1 (1.0)	28 (10.4)	
Non-Response^b^	4 (11.3)	0 (0.0)	9 (4.9)	
Hispanic Ethnicity^a^				0.03
Yes	1 (4.4)	0 (0.0)	14 (6.3)	
No	30 (94.4)	51 (100.0)	244 (90.9)	
Non-Response	1 (1.2)	0 (0.0)	6 (2.7)	
Education Level				0.49
Four-year college degree or higher	11 (34.2)	19 (37.0)	102 (38.0)	
Some college or technical degree (includes Associates)	12 (35.8)	22 (43.9)	117 (45.9)	
High school diploma or GED	8 (27.4)	10 (19.0)	40 (14.8)	
Less than a high school degree	1 (2.6)	0 (0.0)	5 (1.3)	
Employment Status				0.28
Full time work	3 (10.8)	8 (13.6)	55 (22.8)	
Part time or self-employed	3 (10.4)	4 (9.1)	35 (14.1)	
Unemployed/disabled	26 (78.8)	39 (77.4)	174 (63.2)	
Any Unmet Needs^a^^,^^c^	13 (38.5)	13 (25.3)	57 (23.2)	0.20
Lack of Transportation Frequency^a^				0.44
Often	1 (1.1)	4 (7.7)	10 (4.8)	
Sometimes	4 (9.4)	6 (15.4)	29 (12.3)	
Rarely	5 (20.0)	4 (5.7)	40 (14.5)	
Never	22 (69.5)	37 (71.2)	184 (68.2)	
Non-Response	0 (0.0)	0 (0.0)	1 (0.2)	
**Perceived Ease of Use, N (Weighted %)**				
Internet Access by^a^^,^^d^				0.62
Broadband Internet	24 (75.2)	38 (77.5)	216 (81.9)	
Cell Phone Only	1 (4.4)	4 (6.7)	13 (6.2)	
Satellite	4 (11.2)	5 (10.3)	24 (8.5)	
No Internet at home	2 (6.5)	3 (4.7)	11 (3.3)	
Non-Response	1 (3.3)	1 (2.3)	0 (0.0)	
Internet connection speed is sufficient for needs^d^				0.27
Yes	24 (70.6)	43 (85.3)	230 (88.1)	
No	5 (19.6)	4 (7.6)	19 (6.8)	
No internet at home	2 (6.5)	3 (4.7)	11 (3.3)	
Non-Response	1 (3.3)	1 (2.3)	4 (1.8)	
Used a computer at home, at work, or at school^a^	32 (100.0)	46 (89.5)	251 (94.3)	0.03
Has an unlimited data plan.^a^				0.01
Yes	26 (80.5)	40 (81.4)	236 (90.4)	
No	3 (11.8)	8 (14.4)	21 (7.2)	
I don't have a cell phone.	2 (6.6)	0 (0.0)	3 (0.9)	
Non-Response	1 (1.1)	3 (4.3)	4 (1.5)	
**Perceived Usefulness, N (Weighted %)**				
Interested in telemedicine but don't know how to use it^a^				0.86
Yes	5 (17.2)	5 (9.2)	30 (11.4)	
No	26 (80.3)	45 (88.6)	229 (86.7)	
Non-Response	1 (2.5)	1 (2.2)	5 (1.9)	
Used telemedicine before the COVID-19 pandemic^a^				0.001
Yes	6 (15.1)	18 (32.8)	106 (39.2)	
No	26 (84.9)	33 (67.2)	154 (59.7)	
Non-Response	0 (0.0)	0 (0.0)	4 (1.1)	
Had a telemedicine visit by video or telephone with doctor or healthcare professional in past year^e^	20 (61.2)	39 (76.1)	227 (84.9)	0.01
Positive Experience using telemedicine in past year^e^				<0.001
Yes	7 (20.2)	32 (65.7)	222 (82.6)	
No	12 (36.6)	4 (6.1)	4 (1.9)	
No telemedicine visits in past year	12 (38.8)	12 (23.9)	37 (15.1)	
Non-Response	1 (4.4)	3 (4.3)	1 (0.4)	
Comfortable using the telemedicine technology^a^^,e^				<0.001
Yes	13 (37.1)	31 (61.2)	215 (80.4)	
No	7 (24.1)	8 (15.0)	12 (4.5)	
No telemedicine visits in past year	12 (38.8)	12 (23.9)	37 (15.1)	
A telemedicine visit would be easier for me if had				
Training in how to use the technology				0.24
Yes	15 (48.7)	29 (55.4)	111 (44.5)	
No	17 (51.3)	22 (44.6)	149 (53.8)	
Non-Response	0 (0.0)	0 (0.0)	4 (1.7)	
Help getting access to the Internet^a^				0.03
Yes	7 (20.7)	18 (31.7)	69 (25.5)	
No	24 (76.0)	32 (66.5)	195 (74.5)	
Non-Response	1 (3.3)	1 (1.7)	0 (0.0)	
Help getting access to a tablet or other device^a^				0.01
Yes	5 (12.4)	18 (30.5)	72 (26.6)	
No	26 (84.3)	32 (67.5)	192 (73.4)	
Non-Response	1 (3.3)	1 (2.0)	0 (0.0)	
Other^a^				0.68
Yes	2 (4.5)	2 (3.1)	21 (7.1)	
No	29 (92.7)	48 (94.5)	242 (92.1)	
Non-Response	1 (2.8)	1 (2.3)	1 (0.9)	
Communicated with doctor via:				<0.001
Virtually (Email or Video Telemedicine)	10 (38.5)	24 (64.7)	96 (71.8)	
In-Person or Via Telephone Only	13 (61.5)	11 (35.3)	33 (28.2)	

a In univariate ordinal logistic regression models of willingness to participate in a video visit in each health care scenario, this variable met the *a priori* 0.25 significance threshold for at least one scenario and was included in the final multivariate model.

b Non-Response includes don't know and refused responses. In subsequent analyses, for variables with ≥5 non-response answers, a category of “non-response” was included; else non-response was treated as missing.

c This includes at least one of the following: Food, Clothing, Utilities, Childcare, Medicine or Healthcare, Phone, or Other.

d These questions are collinear as the same persons have no internet access at home. A model using each question was assessed and a primary variable selected for the multivariate model.

e These questions are collinear as the same persons indicated no telemedicine visit with a doctor or healthcare professional in the past year. A model using each question was assessed and a primary variable selected for the multivariate model.

Similarly, most were willing to participate in a video telemedicine visit for a non-emergent outpatient MH visit (70.7%) (Supplementary Table 1), or non-emergent health issue requiring a specialty physician (61.6%) (Supplementary Table 2). Those who were willing in the mental health scenario shared similar characteristics as those described in the basic scenario with the addition that those who were willing versus unwilling were more likely to often lack transportation (5.9% vs. 0.0%) but no statistical difference in wanting help accessing the internet was detected. In the specialty care scenario, those who were willing versus unwilling were less likely to be women (9.5% vs. 14.7%), to have any unmet need (20.4% vs. 37.7%) and more likely to have had a positive telemedicine experience in the past year (82.0% vs. 54.4%) and to want help getting access to the internet (28.0% vs. 21.6%).

Respondents compared to non-respondents were more likely to be female (16.9% vs. 12.0%; *p* = 0.01) and white (82.6% vs. 78.1%; *p* = 0.01). (Supplementary Table 3).

**Figure 1:**
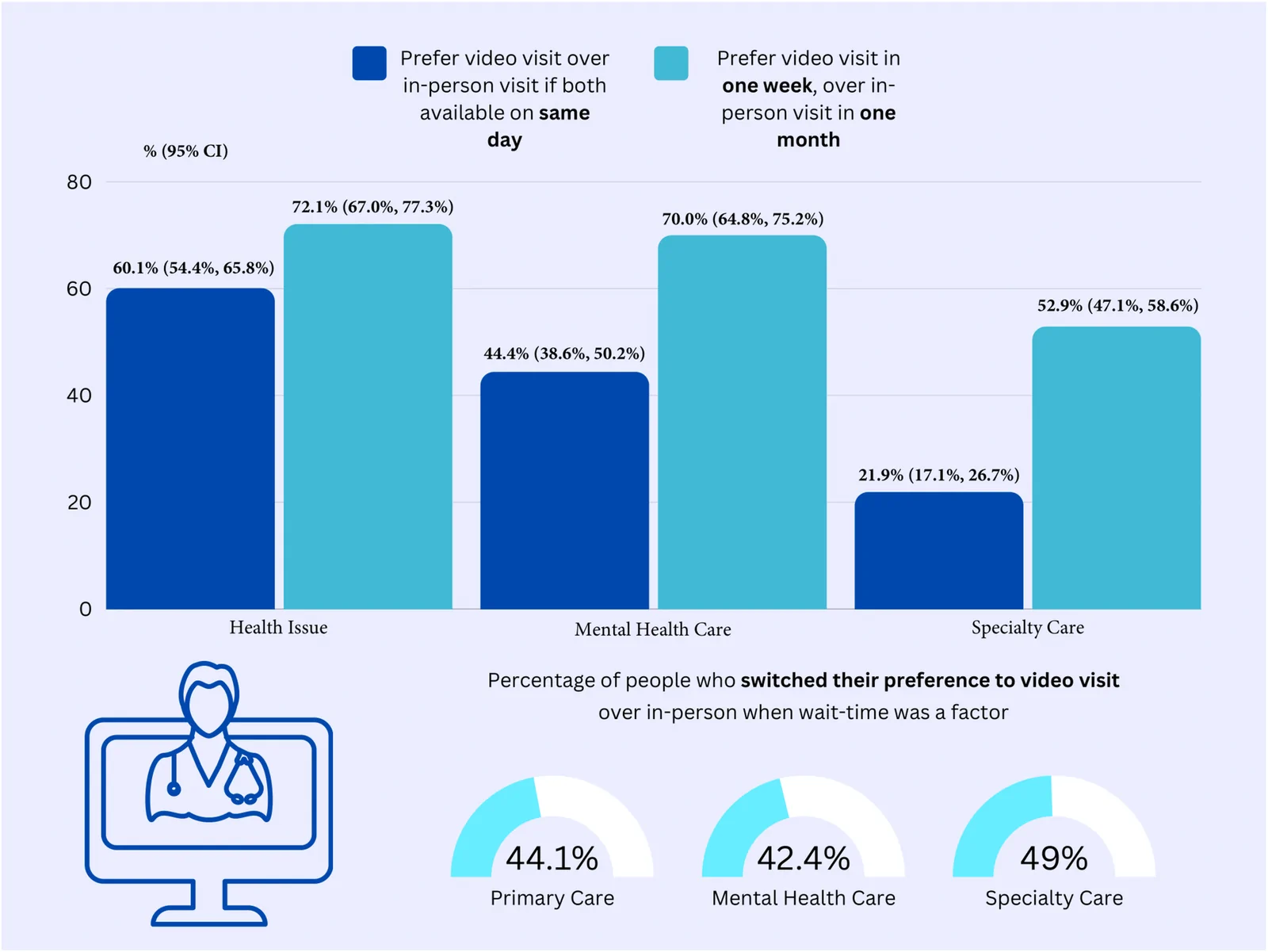
Impact of visit timing on willingness to use video telemedicine.

In all healthcare settings queried, when time was a factor (i.e., a video visit was available in the next week versus an in-person visit in a month), the percentage of veterans who preferred a video visit increased (Figure 1).

### Non-emergent health issue scenario

In adjusted analyses, endorsing being experienced in using telemedicine prior to the pandemic [10.3 percentage points; 95% CI = (2.0, 18.6)], being comfortable using the technology [40.6 percentage points; 95% CI = (22.2, 58.9)], and being interested in telemedicine (i.e., telephone or video) despite not knowing how to use it (versus not endorsing both interest and lack of know-how) [12.0 percentage points, 95% CI = (1.3, 22.8)] had significantly higher probabilities of willingness to use video visits for a non-emergent health issue. (Table 2) Only communicating with their doctor in person or by phone, versus by email or video, was negatively associated with willingness to use video telemedicine [−20.9 percentage points; 95% CI = (−38.7, −3.2)].

**Table 2:** Average marginal effects and 95% confidence intervals for the relationship between external factors, elements of perceived ease of use, and perceived usefulness and willingness to participate in a video visit in three non-emergent healthcare scenarios.

Outcome: Willingness to participate in a video visit with a doctor for …	Adjusted Marginal Effect, % (95% CI, %: LL, UL)		
	Health Issue *N* = 347	Mental Health Outpatient Visit *N* = 341	Health Issue Requiring a Specialty Physician *N* = 344
**Demographics and External Factors**			
Age, 1-Year Increase	−0.4 (−0.8, 0.0)	**−0.6** (**−1.0, −0.3)**^a^	0.0 (−0.4, 0.5)
Comorbidity Score	2.1 (−1.6, 5.9)	0.0 (−4.0, 4.1)	1.1 (−4.4, 6.7)
Urban Residence (Ref: Rural)	3.0 (−6.3, 12.4)	4.8 (−5.0, 14.5)	1.6 (−9.3, 12.5)
Race (Ref: White/Caucasian)			
Black/African American	−7.1 (−24.6, 10.4)	1.9 (−15.1, 18.8)	−2.8 (−22.8, 17.3)
Other	9.7 (−6.0, 25.5)	0.4 (−20.4, 21.1)	11.7 (−6.9, 30.3)
Non-Response^b^	−13.0 (−62.2, 36.2)	−13.2 (−37.5, 11.2)	−23.3 (−57.3, 10.6)
Hispanic Ethnicity (Ref: Not Hispanic)			
Yes	15.2 (−4.7, 35.2)	1.2 (−22.3, 24.6)	8.9 (−24.9, 42.8)
Non-Response^b^	14.7 (−9.4, 38.7)	**25.1** (**10.1, 40.1)**	−15.3 (−76.3, 45.7)
Any Unmet Needs^c^ (Ref: None)	−8.2 (−21.1, 4.6)	−10.7 (−23.0, 1.5)	**−17.0** (**−31.2, −2.7)**
Lack of Transportation Frequency (Ref: Never)			
Often	2.5 (−23.9, 28.8)	**23.4** (**5.6, 41.3)**	16.5 (−14.8, 47.8)
Sometimes	4.6 (−8.7, 17.8)	7.3 (−6.1, 20.7)	14.8 (−0.4, 30.0)
Rarely	9.8 (−2.4, 22.0)	5.6 (−10.7, 21.9)	4.9 (−14.1, 23.8)
**Perceived Ease of Use**			
Internet Access By (Ref: Broadband Internet)			
Cell Phone Only	−9.9 (−32.1, 12.3)	−19.4 (−41.4, 2.6)	−23.3 (−50.0, 3.4)
Satellite	1.2 (−13.8, 16.2)	−17.5 (−37.2, 2.3)	0.0 (−21.3, 21.2)
I have no Internet at home	4.1 (−14.5, 22.7)	4.9 (−14.9, 24.6)	−14.9 (−36.5, 6.7)
Used a computer at home, at work, or at school. (No)	−13.8 (−28.0, 0.5)	9.2 (−15.9, 34.3)	−20.2 (−39.5, −0.9)
Has an unlimited data plan. (Ref: No)			
Yes	8.8 (−6.7, 24.3)	10.0 (−8.0, 28.0)	−1.1 (−18.3, 16.0)
I don't have a cell phone.	−5.5 (−41.8, 30.9)	4.7 (−24.5, 33.8)	**−33.0** (**−60.3, −5.6)**
Non-Response^b^	2.4 (−35.1, 40.0)	−25.6 (−69.6, 18.4)	−25.7 (−64.4, 13.0)
**Perceived Usefulness**			
Interested in telemedicine but don't know how to use it. (Ref: No)			
Yes	**12.0** (**1.3, 22.8)**	**16.6** (**5.3, 28.0)**	**21.1** (**5.9, 36.4)**
Non-Response^b^	−4.6 (−27.3, 18.0)	10.3 (−15.5, 36.1)	4.6 (−34.1, 43.4)
Used telemedicine before the COVID−19 pandemic. (Ref: No)	**10.3** (**2.0, 18.6)**	**11.8** (**2.4, 21.3)**	−2.9 (−13.8, 8.0)
Comfortable using the telemedicine technology. (Ref: No)			
Yes	**40.6** (**22.2, 58.9)**	14.5 (−4.4, 33.4)	**20.6** (**3.6, 37.6)**
No telemedicine visits in past year	**34.8** (**14.9, 54.7)**	7.1 (−14.8, 29.0)	9.9 (−11.6, 31.4)
A telemedicine visit would be easier for me if I had: (Ref: No)			
Help getting access to the Internet	−8.2 (−23.5, 7.1)	−13.4 (−30.2, 3.5)	−2.5 (−23.0, 17.9)
Help getting access to a tablet or other device	11.6 (−1.4, 24.5)	**20.2** (**8.3, 32.1)**	13.5 (−4.8, 31.7)
Other	7.7 (−11.0, 26.5)	10.4 (−8.9, 29.8)	15.3 (−1.5, 32.1)
Communicated with doctor via (Ref: Email or Video Telemedicine)			
In-person or Via Telephone Only	**−20.9** (**−38.7, −3.2)**	−13.4 (−30.0, 3.2)	−11.2 (−28.1, 5.6)

a Bolded differences are significant at the 0.05 level.

b Non-Response includes don't know and refused responses. For variables with ≥5 non-response answers, a category of “non-response” was included; else non-response was treated as missing.

c This includes at least one of the following: Food, Clothing, Utilities, Childcare, Medicine or Healthcare, Phone, or Other.

### Mental health scenario

In adjusted analyses, there was a significantly decreased probability of willingness to use video visits for MH outpatient visits among older patients [−0.6 percentage points per year older; 95% CI = (−1.0, −0.3)]. (Table 2) Often (vs. never) lacking transportation [23.4 percentage points; 95% CI = (5.6, 41.3)], indicating a telemedicine visit would be easier (vs. not easier) if provided help accessing a tablet [20.2 percentage points; 95% CI = (8.3, 32.1)], expressing interest in telemedicine (i.e., telephone or video) despite not knowing how to use it (vs. not endorsing interest and lack of know-how) [16.6 percentage points; 95% CI = (5.3, 28.0)], and using telemedicine (vs. not) prior to the pandemic [11.8 percentage points; 95% CI = (2.4, 21.3)] were associated with differentially higher probabilities of willingness to use MH video visits.

### Specialty care scenario

In adjusted analyses, there was a significantly lower probability of being willing to use video telemedicine for a specialty care visit among those with any unmet need (e.g., food, clothing, utilities, childcare) [−17.0 percentage points; 95% CI = (−31.2, −2.7)] or who reported not having a cell phone [−33.0 percentage points; 95% CI = (−60.3, −5.6)]. Expressing interest in telemedicine (i.e., telephone or video) despite not knowing how to use it (vs. not endorsing both interest and lack of know-how) [21.1 percentage points; 95% CI = (5.9, 36.4)] or being comfortable using the technology [20.6 percentage points; 95% CI = (3.6, 37.6)] were associated with a higher probability of willingness to participate in a video visit for specialty care (Table 2).

Full models are reported in Supplementary Tables 4−6.

### Qualitative results

**Figure 2:**
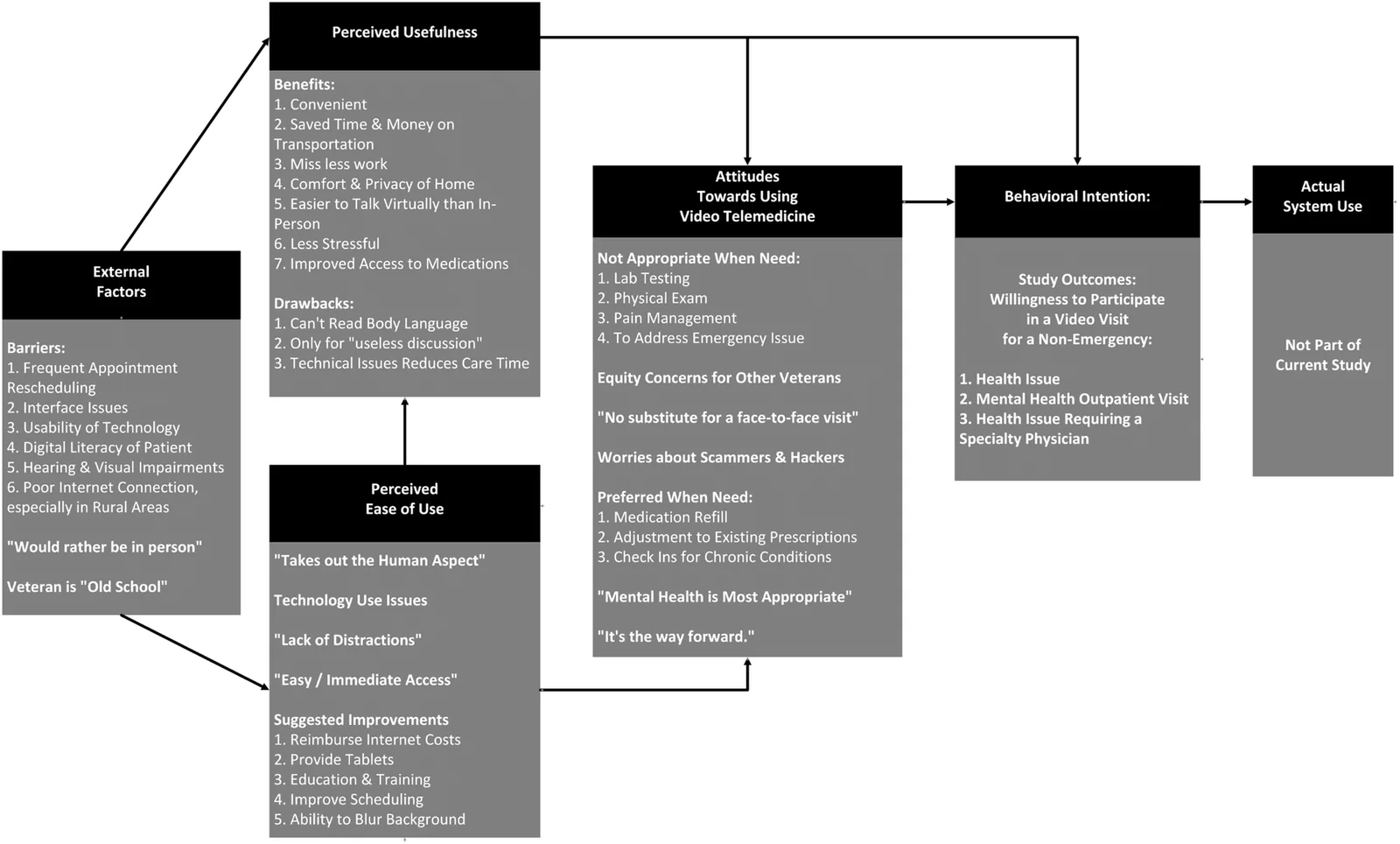
Themes from the open-ended survey items mapped to the Technology Acceptance Model Framework.

Within the technology acceptance model, there are four major themes: 1) external factors affecting, 2) perceived usefulness of, 3) perceived ease of use of, and 4) attitudes towards using video telemedicine (Figure 2) (64). Quotes from open-ended questions representing each theme appear in an abbreviated version in Table 3.

**Table 3:** Example quotes from veteran respondents within the technology acceptance model framework.

Technology Acceptance Model	Sample Statements
External Factors: System and Personal characteristics that affect use of video telemedicine	….For some veterans, telemedicine is more a liability because they don't have good access. And not all veterans are educated enough to use telemedicine appointments, which puts those veterans at a disadvantage and limits their access to basic medical care.
	“I have a smartphone and I don't know how to use it. Same with the computer. I'm very limited in my knowledge in what I'm doing. I'd have to have that all explained.”
	“No, I am old school and prefer in person with my medical doctors because i feel there are things that we can assess in person much better than over online”
Perceived Usefulness: Veteran perception of how useful video telemedicine is	“I don't think telehealth is as good because things like body language is important in a health visit.”
	“Not having to use gas to drive to a doctor’s appointment especially for an issue that can easily be dealt with over the phone.”
	“ease of travel, more likely to arrive on time, it is helpful in limiting exposure to covid because her husband has cancer, it was helpful for counseling appointments, primary care, infectious disease follow up, she finds it extremely useful”
	“It’s very convenient—I can't just pick and choose what days I can come to the VA. Telehealth helps both parties—I don't miss my appointments because of work”
Perceived Ease of Use: The degrees to which the veteran believes using video telemedicine is free of effort	“Would help her out to have someone to teach her how to use the internet and computer. She is unaware how to properly use the internet.”
	“When he gets notifications for his future appointments (telemedicine) he gets scheduling notifications multiple times, and they are repeatedly scheduled and rescheduled constantly. He says the scheduling notification is overwhelming and confusing.”
Attitudes Towards Using Video Telemedicine: Veteran evaluation of the potential consequences of using video telemedicine	“If I'm just getting a prescription refilled or need to adjust my blood pressure medicine, I would rather have a video visit to save me the drive time and waiting at the VA for my appointment. If I am having pain or suicidal thoughts, I need to be seen in person right away and a video call is not appropriate.”
	“Scammers and hackers are one reason why veteran prefers in person visits compared to telemedicine.”
	“I think there is a place for telemedicine but it depends on the situation and appointment type. Can't give a physical examine in telemedicine. Will never replace in person appointments…”

#### Theme 1: external factors

Barriers to using telemedicine included frequent rescheduling of appointments, prohibitive internet costs, and insufficient access to good internet. Sometimes participants had difficulty understanding the provider, especially veterans with hearing or visual impairments. Multiple veterans expressed in-person preferences or viewed themselves as “old school.”

#### Theme 2: perceived usefulness

Many participants indicated video visits were convenient. They felt they could address concerns across a variety of fields (e.g., MH, neurology, dermatology), accomplish care goals, and that replies and follow-up were quickly addressed particularly for lab or test results. Participants appreciated the ability to avoid COVID exposures and worried less about getting themselves or others sick. Some viewed telemedicine as removing appointments that “clog up the system” for veterans needing in-person care more.

The ability of telemedicine to reduce travel burden (i.e., reduced travel times, costs) was often discussed. Veterans viewed telemedicine as a significant benefit for those with mobility or transportation issues, or with caregiver reliance. Others appreciated the ability to keep a telemedicine appointment in circumstances where they would have been unable to keep an in-person appointment (e.g., forgot appointment, during the COVID-19 pandemic, when scheduled provider was unavailable). Telemedicine appointments were also easier to balance with work obligations.

Veterans felt talking to providers via telemedicine was sometimes easier than in-person. Some even felt communicating with their provider over video was just like they were in the room with the same quality of care as an in-person visit, removing anxiety and stress for some and offering others more flexibility. Perceived drawbacks to video visits included an inability to read body language, technical difficulties, and reduction in the time they had to discuss their medical condition.

#### Theme 3: perceived ease of use

Some thought video visits were impersonal or less effective for relationship building. Problems using technology were common. However, participants also reported providers were more attentive, patient, and had fewer distractions during a telemedicine visit. They appreciated telemedicine that facilitated easy access to providers. To improve telemedicine, participants expressed interest in receiving tablets or internet cost reimbursement from VHA. Education and training were also indicated as being necessary and appreciated. Improvements to the scheduling and reminder process or a desire to blur their background when using VVC were endorsed.

#### Theme 4: attitudes towards using video telemedicine

Participants reported feeling telemedicine was not appropriate for all appointment types, including when lab testing, physical exams, or pain management was needed, or to address an emergency issue. Respondents were also concerned about equity for other veterans (e.g., access, costs, disabilities) and information security. Many felt video telemedicine was most appropriate for MH visits. For some, growth in telemedicine was viewed as the way forward.

## Discussion

Despite evidence that video telemedicine is effective, few studies have considered patients’ willingness to participate in video telemedicine (66, 67). In this mixed methods study, we found a large portion of veterans would prefer video telemedicine for a non-emergent health issue, MH, or specialty care visit when time was a factor. Differentially higher rates of willingness to receive care remotely were observed for those expressing interest in telemedicine but not knowing how to use it (all scenarios), using telemedicine prior to the pandemic (health issue and MH care scenarios), and comfort with technology (health issue and specialty care scenarios).

Consistent with prior research, veterans participating in this study appreciated the convenience, time, and financial savings from video visits (33, 68). However, concerns remain about when such visits were appropriate, how to address technological difficulties, and how communication issues could affect the ability to use these services effectively, especially for those with hearing or visual impairments. These concerns align to factors considered by clinicians when deciding to initiate virtual visits, such as patient ability to participate in virtual visits (i.e., internet and technology access; caregiver support, age), patient acuity, clinical goals, or the need for labs or tests (69, 70). Notably, in research regarding patient preferences among veterans issued a VA tablet, those endorsing video visits were more likely to report a “collaborative communication style” with their clinician (34), suggesting communication styles or body language may be relevant in addition to hearing or visual abilities.

As recommended by the veterans in this study and other research efforts, education and technology training would be beneficial (66, 71) and could potentially decrease caregiver reliance. The desire to blur the background during a video visit is consistent with prior work in which patients identified privacy concerns (72). These findings underscore the importance of factors impacting current and subsequent telemedicine experiences, and further, offer opportunities to facilitate increased telemedicine use by addressing education and technology training needs. For routine follow-up visits without a physical exam or laboratory testing, providers should discuss the feasibility of a video visit with the patient to determine their unique needs, preferences, and technology access (73).

Many studies have been conducted to determine who uses telehealth, either by video or telephone. Overall, older, non-white, uninsured, or rural-residing populations underuse telemedicine (5, 74, 75). In our findings, similar to those of Chen et al. (76)*,* patients willing to engage with video telemedicine were more likely to be younger, white, have better connectivity and availability of broadband internet, be more comfortable using technology, and have had previous experience with telemedicine visits (76). It is likely the factors that make people unwilling to participate are interconnected and compound to create a poor experience resulting in less willingness to participate in the future.

The veterans in this study endorsed discomfort with technology and technical difficulties as actionable barriers to video telemedicine use suggesting healthcare systems attempting to increase video telemedicine participation should focus on advocating for and facilitating engagement resources. Focused education to increase technology-based comfort might be beneficial and was recommended by participants in the current and other studies (26, 77) including personalized tutorials using the patient’s own device(s), mailed instructional pamphlets, or practice calls prior to the actual visit (78). Other patient-reported barriers among veterans include not knowing how to schedule a video visit, having prior video visits rescheduled/canceled, or experiencing problems using video visits in the past (79). Efforts to make the first visit successful, such as enacting simple pre-appointment contact protocols, may also positively impact future willingness to use virtual visits.

### Limitations

This research has limitations. First, results may not be generalizable, as the veteran population tends to be older, predominantly male, and white. Further we did not differentiate between urban and rural-residing veterans which may impact the transferability of study findings as rural and urban settings often experience differential access barriers. Additionally, existing VHA infrastructure supported a rapid transition to telemedicine, which might have positively skewed the veteran experience. We note further that this study was unable to assess the gap between behavioral intention and actual use of video visits. Finally, though the study cohort does not represent a random sample, the survey methodologies accounting for the study design remains a study strength despite residual non-response bias.

### Synthesis and future work

Overall, this study showed most veterans are willing to engage with telemedicine for non-urgent health issues, MH, and/or specialty care concerns. A variety of factors, including previous positive experiences with and comfort using telemedicine, were associated with willingness to partake in a video visit. As telemedicine becomes more common, programs addressing underlying patient concerns should be considered. Future work should focus on building patient and provider consensus on when a video visit is medically appropriate, the ways in which healthcare systems can foster and support telemedicine use among providers, and applicable policy improvements supporting both patient and provider choice. Implementing process changes that encourage healthcare teams to discuss the preferred modality of future visits with patients may be a simple but meaningful option.

## Data Availability

Due to US Department of Veterans Affairs (VA) regulations and our ethics agreements, the analytic data sets used for this study are not permitted to leave the VA firewall without a Data Use Agreement. This limitation is consistent with other studies based on VA data. For more information, please visit https://www.virec.research.va.gov or contact theVA Information Resource Center at VIReC@va.gov. Requests to access the datasets should be directed to Amy O’Shea, amy.oshea@va.gov.
