# Supplementary material for: Willingness for video visits for non-emergent health issues, mental health, or specialty visits
Source: Front Digit Health. 2026 Aug 27;8:1845428. doi: 10.3389/fdgth.2026.1845428 (PMC13559756; doi:10.3389/fdgth.2026.1845428)
Supplement: Supplementary file 1 [file Supplementaryfile1.docx]

**Supplementary Files**

Willingness to Use Video Visits for Non-Emergent Health Issues, Mental Health, or Specialty Visits

**Table of Contents**

**eTable 1.** Counts and weighted percentages for survey responses, stratified by veteran willingness to participate in a video telemedicine visit for a non-emergent mental health visit.

**eTable 2.** Counts and weighted percentages for survey responses, stratified by veteran willingness to participate in a video telemedicine visit for a non-emergent specialty care visit.

**eTable 3.** Patient characteristics of respondents and non-respondents.

**eTable 4.** Average marginal effects and 95% confidence intervals for the relationship between external factors, elements of perceived ease of use, and perceived usefulness and willingness to participate in a video visit with a doctor for a non-emergent health issue.

**eTable5.** Average marginal effects and 95% confidence intervals for the relationship between external factors, elements of perceived ease of use, and perceived usefulness and willingness to participate in a video visit with a doctor for a non-emergent mental health outpatient visit.

**eTable 6.** Average marginal effects and 95% confidence intervals for the relationship between external factors, elements of perceived ease of use, and perceived usefulness and willingness to participate in a video visit with a doctor for a health issue requiring a specialty physician.

**Appendix Table 1. Counts and weighted percentages for survey responses, stratified by veteran willingness to participate in a video telemedicine visit for a non-emergent mental health visit.**

| **Survey Question** | **Unwilling**  **N= 60** | **Neutral**  **N = 40** | **Willing**  **N = 241** | **p-value** |
| --- | --- | --- | --- | --- |
| **Demographics and External Factors, N (Weighted %)** | | | |  |
| Age^a^, Mean (95% CI) | 65.6  (61.3, 69.9) | 66.3  (61.8, 70.9) | 59.3  (57.3, 61.2) | 0.002 |
| Comorbidity Score, Mean (95% CI) | 0.6  (0.4, 0.9) | 0.6  (0.3, 0.9) | 0.6  (0.4, 0.7) | 0.90 |
| Residential Rurality |  |  |  | 0.04 |
| Urban | 30 (64.7) | 15 (48.5) | 123 (69.6) |  |
| Rural | 30 (35.5) | 25 (51.5) | 118 (30.4) |  |
| Female Gender | 9 (11.4) | 6 (10.8) | 44 (13.8) | 0.79 |
| Race^a^ |  |  |  | 0.78 |
| Black/African American | 4 (8.8) | 1 (5.0) | 19 (10.6) |  |
| White/Caucasian | 51 (82.6) | 35 (83.7) | 188 (74.3) |  |
| Other | 2 (4.1) | 2 (5.4) | 26 (10.2) |  |
| Non-Response^b^ | 3 (4.5) | 2 (5.9) | 8 (4.9) |  |
| Hispanic Ethnicity^a^ |  |  |  | 0.32 |
| Yes | 2 (3.1) | 1 (2.1) | 11 (5.6) |  |
| No | 57 (96.2) | 39 (97.9) | 225 (91.9) |  |
| Non-Response | 1 (0.7) | 0 (0.0) | 5 (2.5) |  |
| Education Level |  |  |  | 0.75 |
| Four-year college degree or higher | 21 (36.3) | 14 (33.0) | 93 (37.5) |  |
| Some college or technical degree (includes Associates) | 25 (39.5) | 20 (51.6) | 105 (45.6) |  |
| High school diploma or GED | 13 (22.8) | 6 (15.4) | 38 (15.6) |  |
| Less than a high school degree | 1 (1.5) | 0 (0.0) | 5 (1.4) |  |
| Employment Status |  |  |  | 0.09 |
| Full time work | 9 (13.6) | 4 (7.4) | 52 (23.8) |  |
| Part time or self-employed | 7 (13.2) | 4 (10.2) | 30 (12.9) |  |
| Unemployed/disabled | 44 (73.1) | 32 (82.4) | 159 (63.3) |  |
| Any Unmet Needs^a,c^ | 19 (29.5) | 9 (24.0) | 53 (24.0) | 0.70 |
| Lack of Transportation Frequency^a^ |  |  |  | 0.02 |
| Often | 0 (0.0) | 3 (6.5) | 12 (5.9) |  |
| Sometimes | 6 (9.7) | 5 (13.9) | 27 (13.0) |  |
| Rarely | 14 (24.6) | 2 (3.1) | 33 (13.3) |  |
| Never | 40 (65.7) | 30 (76.5) | 168 (67.5) |  |
| Non-Response | 0 (0.0) | 0 (0.0) | 1 (0.2) |  |
| **Perceived Ease of Use, N (Weighted %)** | | | |  |
| Internet Access by^a,d^ |  |  |  | 0.24 |
| Broadband Internet | 43 (70.5) | 34 (85.7) | 198 (83.4) |  |
| Cell Phone Only | 3 (6.7) | 2 (4.6) | 12 (5.5) |  |
| Satellite | 9 (15.1) | 2 (4.8) | 20 (7.7) |  |
| I have no Internet at home | 5 (7.7) | 1 (2.4) | 10 (3.2) |  |
| Non-Response | 0 (0.0) | 1 (2.4) | 1 (0.2) |  |
| Internet connection speed is sufficient for needs.^d^ |  |  |  | 0.09 |
| Yes | 46 (74.0) | 36 (89.7) | 209 (87.8) |  |
| No | 6 (12.0) | 2 (5.2) | 20 (8.0) |  |
| No internet at home | 5 (7.7) | 1 (2.4) | 10 (3.2) |  |
| Non-Response | 9 (6.3) | 1 (2.7) | 2 (1.0) |  |
| Used a computer at home, at work, or at school^a^ | 57 (93.6) | 36 (90.4) | 231 (95.7) | 0.40 |
| Has an unlimited data plan.^a^ |  |  |  | 0.17 |
| Yes | 50 (83.4) | 32 (80.9) | 216 (91.4) |  |
| No | 6 (10.2) | 4 (11.5) | 20 (6.9) |  |
| I don’t have a cell phone. | 2 (2.7) | 3 (5.4) | 3 (1.2) |  |
| Non-Response | 4 (4.0) | 2 (2.4) | 2 (1.0) |  |
| **Perceived Usefulness, N (Weighted %)** | | | |  |
| Interested in telemedicine but don’t know how to use it^a^ |  |  |  | 0.93 |
| Yes | 6 (9.7) | 6 (15.8) | 28 (11.9) |  |
| No | 52 (87.3) | 33 (81.5) | 208 (85.9) |  |
| Non-Response | 2 (2.9) | 1 (2.8) | 5 (2.2) |  |
| Used telemedicine before the COVID-19 pandemic^a^ |  |  |  | 0.003 |
| Yes | 13 (18.9) | 13 (32.8) | 100 (40.2) |  |
| No | 46 (79.1) | 27 (67.2) | 139 (59.5) |  |
| Non-Response | 1 (2.0) | 0 (0.0) | 2 (0.3) |  |
| Had a telemedicine visit by video or telephone with doctor or healthcare professional in past year^e^ | 43 (68.5) | 29 (72.5) | 209 (85.8) | 0.006 |
| Positive Experience using telemedicine in past year^e^ |  |  |  | <0.001 |
| Yes | 29 (44.3) | 24 (61.9) | 203 (83.0) |  |
| No | 11 (19.0) | 5 (10.6) | 4 (2.1) |  |
| Had no telemedicine visit in past year | 17 (31.5) | 11 (27.5) | 32 (14.2) |  |
| Non-Response | 3 (5.3) | 0 (0.0) | 2 (0.7) |  |
| Comfortable using the telemedicine technology^a,e^ |  |  |  | 0.002 |
| Yes | 34 (54.2) | 25 (62.3) | 195 (79.6) |  |
| No | 9 (14.3) | 4 (10.1) | 14 (6.2) |  |
| Had no telemedicine visit in past year | 17 (31.5) | 11 (27.5) | 32 (14.2) |  |
| A Telemedicine visit would be easier for me if I had |  |  |  |  |
| Training in how to use the technology |  |  |  | 0.91 |
| Yes | 27 (44.6) | 18 (49.9) | 109 (47.4) |  |
| No | 32 (53.7) | 21 (47.3) | 130 (51.5) |  |
| Non-Response | 1 (1.7) | 1 (2.8) | 2 (1.1) |  |
| Help getting access to the Internet^a^ | 14 (23.0) |  |  | 0.11 |
| Yes | 14 (23.0) | 13 (32.3) | 66 (25.7) |  |
| No | 45 (75.1) | 26 (65.5) | 175 (74.3) |  |
| Non-Response | 1 (1.9) | 1 (2.2) | 0 (0.0) |  |
| Help getting access to a tablet or other device^a^ |  |  |  | 0.05 |
| Yes | 11 (17.6) | 12 (27.1) | 70 (27.7) |  |
| No | 48 (80.5) | 27 (70.3) | 171 (72.3) |  |
| Non-Response | 1 (1.9) | 1 (2.5) | 0 (0.0) |  |
| Other^a^ |  |  |  | 0.86 |
| Yes | 3 (4.0) | 3 (6.5) | 19 (7.0) |  |
| No | 56 (94.1) | 36 (91.1) | 221 (92.0) |  |
| Non-Response | 1 (1.9) | 1 (2.4) | 1 (0.9) |  |
| Communicated with doctor via: |  |  |  | <0.001 |
| Virtually (Email or Video Telemedicine) | 42 (64.4) | 29 (70.8) | 213 (87.5) |  |
| In-Person or Via Telephone Only | 18 (35.6) | 11 (29.2) | 28 (12.5) |  |

a. In univariate ordinal logistic regression models of willingness to participate in a video visit in each health care scenarios, this variable met the a priori 0.25 significance threshold for at least one scenario and was included in the final multivariate model.
b. Non-Response includes don’t know and refused responses. In subsequent analyses, for variables with ≥5 non-response answers, a category of “non-response” was included; else non-response was treated as missing.
c. This includes at least one of the following: Food, Clothing, Utilities, Childcare, Medicine or Healthcare, Phone, or Other
d. These questions are collinear as the same persons have no internet access at home. A model using each question was assessed and a primary variable selected for the multivariate model.
e. These questions are collinear as the same persons did not have a telemedicine visit by video or phone with a doctor or healthcare professional in the past year. A model using each question was assessed and a primary variable selected for the multivariate model.

**Appendix Table 2. Counts and weighted percentages for survey responses, stratified by veteran willingness to participate in a video telemedicine visit for a non-emergent specialty care visit. ^a^**

| **Survey Question** | **Unwilling**  **N= 79** | **Neutral**  **N = 53** | **Willing**  **N = 212** | **p-value** |
| --- | --- | --- | --- | --- |
| **Demographics and External Factors, N (Weighted %)** | | | |  |
| Age^a^, Mean (95% CI) | 60.3  (56.1, 64.5) | 62.1  (58.2, 66.1) | 61.2  (59.2, 63.2) | 0.82 |
| Comorbidity Score, Mean (95% CI) | 05  (0.3, 0.7) | 0.4  (0.2, 0.6) | 0.7  (0.5, 0.8) | 0.17 |
| Residential Rurality |  |  |  | 0.66 |
| Urban | 41 (68.4) | 25 (61.2) | 105 (67.4) |  |
| Rural | 38 (31.6) | 28 (38.8) | 107 (32.6) |  |
| Female Gender | 17 (14.7) | 14 (23.4) | 27 (9.5) | 0.03 |
| Race^a^ |  |  |  | 0.13 |
| Black/African American | 12 (13.8) | 3 (7.5) | 19 (9.4) |  |
| White/Caucasian | 57 (70.1) | 51 (84.3) | 177 (76.5) |  |
| Other | 22 (5.7) | 2 (3.6) | 27 (11.3) |  |
| Non-Response^b^ | 8 (10.4) | 7 (4.6) | 9 (2.8) |  |
| Hispanic Ethnicity^a^ |  |  |  | 0.25 |
| Yes | 3 (5.4) | 2 (4.0) | 10 (5.6) |  |
| No | 72 (89.8) | 51 (96.0) | 200 (93.2) |  |
| Non-response | 4 (4.9) | 0 (0.0) | 2 (1.2) |  |
| Education Level |  |  |  | 0.90 |
| Four-year college degree or higher | 32 (38.9) | 22 (40.6) | 75 (35.2) |  |
| Some college or technical degree (includes Associates) | 34 (41.5) | 21 (43.6) | 97 (47.3) |  |
| High school diploma or GED | 12 (18.6) | 10 (15.9) | 35 (15.9) |  |
| Less than a high school degree | 1 (1.1) | 0 (0.0) | 5 (1.6) |  |
| Employment Status |  |  |  | 0.69 |
| Full time work | 13 (16.8) | 12 (24.3) | 41 (21.1) |  |
| Part time or self-employed | 8 (11.6) | 8 (17.4) | 25 (12.1) |  |
| Unemployed/disabled | 58 (71.6) | 33 (58.4) | 146 (66.9) |  |
| Any Unmet Needs^a,c^ | 28 (37.7) | 14 (23.6) | 40 (20.4) | 0.02 |
| Lack of Transportation Frequency^a^ |  |  |  | 0.24 |
| Often | 3 (3.7) | 0 (0.0) | 12 (6.5) |  |
| Sometimes | 9 (10.6) | 5 (11.4) | 25 (13.6) |  |
| Rarely | 14 (17.7) | 7 (8.9) | 27 (13.0) |  |
| Never | 53 (68.0) | 41 (79.7) | 147 (66.6) |  |
| Non-response | 0 (0.0) | 0 (0.0) | 1 (0.2) |  |
| **Perceived Ease of Use, N (Weighted %)** | | | |  |
| Internet Access by^a,d^ |  |  |  | 0.56 |
| Broadband Internet | 63 (74.7) | 42 (83.3) | 171 (82.5) |  |
| Cell Phone Only | 4 (8.1) | 3 (5.7) | 11 (5.5) |  |
| Satellite | 8 (11.6) | 4 (4.8) | 21 (9.2) |  |
| I have no Internet at home | 3 (4.5) | 4 (6.1) | 8 (2.6) |  |
| Non-response | 1 (1.1) | 0 (0.0) | 1 (0.2) |  |
| Internet connection speed is sufficient for needs.^d^ |  |  |  | 0.33 |
| Yes | 67 (81.4) | 43 (84.3) | 185 (88.4) |  |
| No | 6 (9.6) | 6 (9.6) | 16 (7.4) |  |
| No internet at home | 3 (4.5) | 4 (6.1) | 8 (2.6) |  |
| Non-response | 3 (4.5) | 0 (0.0) | 3 (1.6) |  |
| Used a computer at home, at work, or at school.^a^ | 77 (96.6) | 51 (96.4) | 199 (93.1) | 0.46 |
| Has an unlimited data plan.^a^ |  |  |  | 0.44 |
| Yes | 67 (86.5) | 45 (86.2) | 188 (89.8) |  |
| No | 6 (6.8) | 5 (10.9) | 20 (8.3) |  |
| I don’t have a cell phone. | 10 (2.7) | 5 (0.5) | 8 (0.9) |  |
| Non-response | 4 (4.0) | 2 (2.4) | 2 (1.0) |  |
| **Perceived Usefulness, N (Weighted %)** | | | |  |
| Interested in telemedicine but don’t know how to use it^a^ |  |  |  | 0.16 |
| Yes | 6 (8.6) | 4 (6.4) | 30 (14.3) |  |
| No | 70 (88.0) | 49 (93.6) | 177 (83.2) |  |
| Non-Response | 3 (3.4) | 0 (0.0) | 5 (2.5) |  |
| Used telemedicine before the COVID-19 pandemic |  |  |  | 0.57 |
| Yes | 29 (34.9) | 23 (40.3) | 76 (34.9) |  |
| No | 50 (65.1) | 30 (59.7) | 133 (64.2) |  |
| Non-Response | 0 (0.0) | 0 (0.0) | 3 (0.9) |  |
| Had a telemedicine visit by video or telephone with doctor or healthcare professional in past year^e^ | 60 (73.8) | 43 (81.5) | 182 (84.9) | 0.12 |
| Positive Experience using telemedicine in past year^e^ |  |  |  | <0.001 |
| Yes | 44 (54.4) | 39 (75.4) | 177 (82.0) |  |
| No | 12 (14.4) | 3 (4.8) | 5 (2.9) |  |
| Had no telemedicine visit in past year | 19 (26.2) | 10 (18.5) | 30 (15.1) |  |
| Non-response | 4 (5.0) | 1 (1.3) | 0 (0.0) |  |
| Comfortable using the telemedicine technology^a,e^ |  |  |  | 0.13 |
| Yes | 54 (65.4) | 35 (68.1) | 169 (78.5) |  |
| No | 6 (8.4) | 8 (13.4) | 13 (6.4) |  |
| Had no telemedicine visit in past year | 19 (26.2) | 10 (18.5) | 30 (15.1) |  |
| A Telemedicine visit would be easier for me if I had^a^ |  |  |  |  |
| Training in how to use the technology |  |  |  |  |
| Yes | 33 (43.1) | 22 (41.9) | 101 (50.0) | 0.46 |
| No | 46 (56.9) | 30 (56.0) | 109 (48.8) |  |
| Non-response | 0 (0.0) | 1 (2.1) | 2 (1.2) |  |
| Help getting access to the Internet^a^ |  |  |  | 0.01 |
| Yes | 19 (21.6) | 14 (25.4) | 61 (28.0) |  |
| No | 58 (76.0) | 39 (74.6) | 151 (72.0) |  |
| Non-response | 2 (2.4) | 0 (0.0) | 0 (0.0) |  |
| Help getting access to a tablet or other device^a^ |  |  |  | 0.51 |
| Yes | 17 (20.1) | 17 (28.3) | 60 (27.2) |  |
| No | 61 (78.5) | 36 (71.7) | 151 (72.4) |  |
| Non-response | 1 (1.4) | 0 (0.0) | 1 (0.5) |  |
| Other^a^ |  |  |  | 0.21 |
| Yes | 3 (2.1) | 4 (7.5) | 18 (7.7) |  |
| No | 74 (95.4) | 49 (92.5) | 193 (91.2) |  |
| Non-response | 3 (2.1) | 4 (7.5) | 18 (7.7) |  |
| Communicated with doctor via: |  |  |  | 0.17 |
| Virtually (Email or Video Telemedicine) | 25 (54.4) | 24 (67.9) | 80 (70.2) |  |
| In-Person or Via Telephone Only | 18 (45.6) | 9 (32.1) | 30 (29.8) |  |

a. In univariate ordinal logistic regression models of willingness to participate in a video visit in each health care scenarios, this variable met the a priori 0.25 significance threshold for at least one scenario and was included in the final multivariate model.
b. Non-Response includes don’t know and refused responses. In subsequent analyses, for variables with ≥5 non-response answers, a category of “non-response” was included; else non-response was treated as missing.
c. This includes at least one of the following: Food, Clothing, Utilities, Childcare, Medicine or Healthcare, Phone, or Other
d. These questions are collinear as the same persons have no internet access at home. A model using each question was assessed and a primary variable selected for the multivariate model.
e. These questions are collinear as the same persons did not have a telemedicine visit by video or phone with a doctor or healthcare professional in the past year. A model using each question was assessed and a primary variable selected for the multivariate model.

**Appendix Table 3. Patient characteristics of respondents and non-respondents.**

|  | **CATI Completed** | |  |
| --- | --- | --- | --- |
| **Demographic** | **Yes**  **N=350** | **No**  **N=2495** | **p-value** |
| Age, Mean (95% CI) | 61.9 | 61.2 | 0.446 |
| Comorbidity Score, Mean (95% CI) | 0.6 | 0.5 | 0.132 |
| Gender, N(%) |  |  | 0.010 |
| Female | 59 (16.9) | 299 (12.0) |  |
| Male | 291 (83.1) | 2196 (88.0) |  |
| Race |  |  | 0.010 |
| Black/African American | 34 (9.7) | 371 (14.9) |  |
| White/Caucasian | 289 (82.6) | 1948 (78.1) |  |
| Other | 10 (2.9) | 34 (1.4) |  |
| Non-Response | 17 (4.9) | 142 (5.7) |  |
| Hispanic Ethnicity |  |  | 0.382 |
| Yes | 9 (2.6) | 97 (3.9) |  |
| No | 294 (84.0) | 2098 (84.1) |  |
| Non-Response | 47 (13.4) | 300 (12.0) |  |

**Appendix Table 4. Average marginal effects and 95% confidence intervals for the relationship between external factors, elements of perceived ease of use, and perceived usefulness and willingness to participate in a video visit with a doctor for a non-emergent health issue.**

|  | **Model 1: Non-Emergency Health Issue** | | |
| --- | --- | --- | --- |
|  | **Adjusted Marginal Effect, % (95% CI, %: LL, UL)** | | |
| **Demographics and External Factors** | **Unwilling** | **Neutral** | **Willing** |
| Age, 1-Year Increase | 0.2 (0.0, 0.4) | 0.2 (0.0, 0.4) | -0.4 (-0.8, 0.0) |
| Comorbidity Score, 1-Unit Increase | -1.1 (-3.1, 0.8) | -1.0 (-2.8, 0.8) | 2.1 (-1.6, 5.9) |
| Urban Residence (Ref: Rural) | -1.6 (-6.4, 3.3) | -1.5 (-6.0, 3.0) | 3.0 (-6.3, 12.4) |
| Race (Ref: White/Caucasian) |  |  |  |
| Black/African American | 3.8 (-6.0, 13.6) | 3.3 (-4.5, 11.0) | -7.1 (-24.6, 10.4) |
| Other | -4.6 (-11.4, 2.2) | -5.1 (-14.2, 4.0) | 9.7 (-6.0, 25.5) |
| Non-Response^a^ | 7.4 (-23.5, 38.2) | 5.6 (-12.8, 24.1) | -13.0 (-62.2, 36.2) |
| Hispanic Ethnicity (Ref: Not Hispanic) |  |  |  |
| Yes | -7.0 (-15.0, 1.0) | -8.2 (-20.4, 4.1) | 15.2 (-4.7, 35.2) |
| Non-Response^a^ | -6.8 (-16.9, 3.3) | -7.8 (-22.1, 6.4) | 14.7 (-9.4, 38.7) |
| Any Unmet Needs^b^ (Ref: None) | 4.4 (-2.9, 11.7) | 3.8 (-1.9, 9.5) | -8.2 (-21.1, 4.6) |
| Lack of Transportation Frequency  (Ref: Never) |  |  |  |
| Often | -1.3 (-14.9, 12.3) | -1.2 (-13.9, 11.6) | 2.5 (-23.9, 28.8) |
| Sometimes | -2.4 (-9.1, 4.4) | -2.2 (-8.7, 4.3) | 4.6 (-8.7, 17.8) |
| Rarely | -4.9 (-10.7, 1.0) | -4.9 (-11.5, 1.6) | 9.8 (-2.4, 22.0) |
| **Perceived Ease of Use** |  |  |  |
| Internet Access By  (Ref: Broadband Internet) |  |  |  |
| Cell Phone Only | 5.5 (-7.9, 18.9) | 4.3 (-4.5, 13.2) | -9.9 (-32.1, 12.3) |
| Satellite | -0.6 (-8.3, 7.1) | -0.6 (-7.9, 6.7) | 1.2 (-13.8, 16.2) |
| I have no Internet at home | -2.1 (-11.2, 7.1) | -2.0 (-11.5, 7.4) | 4.1 (-14.5, 22.7) |
| Used a computer at home, at work, or at school. (Ref: No) | **6.6 (-0.1, 13.3)^c^** | 7.2 (-0.8, 15.1) | -13.8 (-28.0, 0.5) |
| Has an unlimited data plan. (Ref: No) |  |  |  |
| Yes | -4.8 (-13.8, 4.2) | -4.0 (-10.6, 2.6) | 8.8 (-6.7, 24.3) |
| I don’t have a cell phone. | 3.3 (-19.4, 26.1) | 2.1 (-11.5, 15.8) | -5.5 (-41.8, 30.9) |
| Non-Response^a^ | -1.4 (-22.6, 19.8) | -1.0 (-17.4, 15.3) | 2.4 (-35.1, 40.0) |
| **Perceived Usefulness** |  |  |  |
| Interested in telemedicine but don’t know how to use it. (Ref: No) |  |  |  |
| Yes | **-6.0 (-11.1, -0.8)** | **-6.1 (-12.0, -0.1)** | **12.0 (1.3, 22.8)** |
| Non-Response^a^ | 2.6 (-10.4, 15.7) | 2.0 (-7.6, 11.6) | -4.6 (-27.3, 18.0) |
| Used telemedicine before the COVID-19 pandemic. (Ref: No) | **-5.1 (-9.3, -0.9)** | **-5.2 (-9.6, -0.8)** | **10.3 (2.0, 18.6)** |
| Comfortable using the telemedicine technology. (Ref: No) |  |  |  |
| Yes | **-27.1 (-43.7, -10.6)** | **-13.4 (-18.8, -8.1)** | **40.6 (22.2, 58.9)** |
| No telemedicine visits in past year | **-24.4 (-41.2, -7.5)** | **-10.4 (-17.0, -3.9)** | **34.8 (14.9, 54.7)** |
| A telemedicine visit would be easier for me if I had: (Ref: No) |  |  |  |
| Help getting access to the Internet | 4.4 (-4.2, 12.9) | 3.8 (-3.1, 10.6) | -8.2 (-23.5, 7.1) |
| Help getting access to a tablet or other device | -5.8 (-12.5, 0.8) | -5.7 (-12.3, 0.9) | 11.6 (-1.4, 24.5) |
| Other | -3.8 (-12.4, 4.8) | -4.0 (-14.1, 6.2) | 7.7 (-11.0, 26.5) |
| Communicated with doctor via  (Ref: Email or Video Telemedicine) |  |  |  |
| In-person or Via Telephone Only | **11.7 (0.3, 23.0)** | **9.3 (2.2, 16.4)** | **-20.9 (-38.7, -3.2)** |

a. Non-Response includes don’t know and refused responses. For variables with ≥5 non-response answers, a category of “non-response” was included; else non-response was treated as missing.
b. This includes at least one of the following: Food, Clothing, Utilities, Childcare, Medicine or Healthcare, Phone, or Other
c. Bolded differences are significant at the 0.05 level

**Appendix Table 5. Average marginal effects and 95% confidence intervals for the relationship between external factors, elements of perceived ease of use, and perceived usefulness and willingness to participate in a video telemedicine visits in a non-emergent mental health outpatient visit.**

|  | **Model 2: Mental Health Outpatient Visit** | | |
| --- | --- | --- | --- |
|  | **Adjusted Marginal Effect, % (95% CI, %: LL, UL)** | | |
| **Demographics and External Factors** | **Unwilling** | **Neutral** | **Willing** |
| Age, 1-Year Increase | **0.5 (0.2, 0.8)^a^** | **0.2 (0.1, 0.3)** | **-0.6 (-1.0, -0.3)** |
| Comorbidity Score, 1-Unit Increase | 0.0 (-4.0, 4.1) | 0.0 (-1.0, 0.9) | 0.0 (-4.0, 4.1) |
| Urban Residence (Ref: Rural) | 4.8 (-5.0, 14.5) | -1.1 (-3.4, 1.2) | 4.8 (-5.0, 14.5) |
| Race (Ref: White/Caucasian) |  |  |  |
| Black/African American | -1.4 (-14.1, 11.3) | -0.5 (-4.7, 3.8) | 1.9 (-15.1, 18.8) |
| Other | -0.3 (-16.1, 15.5) | -0.1 (-5.1, 4.9) | 0.4 (-20.4, 21.1) |
| Non-Response^b^ | 10.7 (-10.3, 31.7) | 2.4 (-1.1, 6.0) | -13.2 (-37.5, 11.2) |
| Hispanic Ethnicity (Ref: Not Hispanic) |  |  |  |
| Yes | -0.9 (-18.6, 16.9) | -0.3 (-6.0, 5.4) | 1.2 (-22.3, 24.6) |
| Non-Response^b^ | **-16.8 (-25.9, -7.8)** | **-8.2 (-14.9, -1.5)** | **25.1 (10.1, 40.1)** |
| Any Unmet Needs^c^ (Ref: None) | 8.5 (-1.5, 18.5) | 2.2 (-0.2, 4.7) | -10.7 (-23.0, 1.5) |
| Lack of Transportation Frequency  (Ref: Never) |  |  |  |
| Often | **-16.3 (-27.7, -4.8)** | **-7.2 (-14.1, -0.3)** | **23.4 (5.6, 41.3)** |
| Sometimes | -5.5 (-15.4, 4.4) | -1.8 (-5.4, 1.8) | 7.3 (-6.1, 20.7) |
| Rarely | -4.3 (-16.4, 7.8) | -1.4 (-5.6, 2.9) | 5.6 (-10.7, 21.9) |
| **Perceived Ease of Use** |  |  |  |
| Internet Access By (Ref: Broadband Internet) |  |  |  |
| Cell Phone Only | 16.0 (-3.9, 35.8) | **3.4 (0.8, 6.1)** | -19.4 (-41.4, 2.6) |
| Satellite | 14.2 (-3.2, 31.6) | **3.2 (0.5, 6.0)** | -17.5 (-37.2, 2.3) |
| I have no Internet at home | -3.5 (-17.6, 10.5) | -1.3 (-7.0, 4.4) | 4.9 (-14.9, 24.6) |
| Used a computer at home, at work, or at school. (Ref: No) | -7.3 (-28.1, 13.4) | -1.9 (-6.4, 2.6) | 9.2 (-15.9, 34.3) |
| Has an unlimited data plan. (Ref: No) |  |  |  |
| Yes | -7.9 (-22.7, 6.9) | -2.1 (-5.4, 1.2) | 10.0 (-8.0, 28.0) |
| I don’t have a cell phone. | -3.8 (-27.2, 19.6) | -0.9 (-6.7, 4.9) | 4.7 (-24.5, 33.8) |
| Non-Response^b^ | 24.2 (-21.7, 70.1) | 1.4 (-2.9, 5.8) | -25.6 (-69.6, 18.4) |
| **Perceived Usefulness** |  |  |  |
| Interested in telemedicine but don’t know how to use it. (Ref: No) |  |  |  |
| Yes | **-12.1 (-20.2, -4.1)** | **-4.5 (-8.2, -0.8)** | **16.6 (5.3, 28.0)** |
| Non-Response^b^ | -7.8 (-26.4, 10.8) | -2.5 (-9.8, 4.7) | 10.3 (-15.5, 36.1) |
| Used telemedicine before the COVID-19 pandemic. (Ref: No) | **-8.8 (-15.9, -1.8)** | **-3.0 (-5.7, -0.2)** | **11.8 (2.4, 21.3)** |
| Comfortable using the telemedicine technology. (Ref: No) |  |  |  |
| Yes | -11.5 (-27.5, 4.5) | -3.0 (-6.2, 0.2) | 14.5 (-4.4, 33.4) |
| No telemedicine visits in past year | -5.9 (-24.2, 12.5) | -1.3 (-4.9, 2.4) | 7.1 (-14.8, 29.0) |
| A telemedicine visit would be easier for me if I had: (Ref: No) |  |  |  |
| Help getting access to the Internet | 10.7 (-3.3, 24.6) | 2.7 (-0.4, 5.8) | -13.4 (-30.2, 3.5) |
| Help getting access to a tablet or other device | **-15.0 (-23.8, -6.2)** | **-5.2 (-8.9, -1.5)** | **20.2 (8.3, 32.1)** |
| Other | -7.6 (-21.0, 5.9) | -2.8 (-8.8, 3.2) | 10.4 (-8.9, 29.8) |
| Communicated with doctor via  (Ref: Email or Video Telemedicine) |  |  |  |
| In-person or Via Telephone Only | **10.6 (-3.0, 24.3)** | **2.8 (-0.3, 6.0)** | -13.4 (-30.0, 3.2) |

a. Bolded differences are significant at the 0.05 level
b. Non-Response includes don’t know and refused responses. For variables with ≥5 non-response answers, a category of “non-response” was included; else non-response was treated as missing.
c. This includes at least one of the following: Food, Clothing, Utilities, Childcare, Medicine or Healthcare, Phone, or Other

**Appendix Table 6. Average marginal effects and 95% confidence intervals for the relationship between external factors, elements of perceived ease of use, and perceived usefulness and willingness to participate in a video telemedicine visits for a non-emergent health issue requiring a specialist.**

|  | **Model 3: Specialty Care Setting** | | |
| --- | --- | --- | --- |
|  | **Adjusted Marginal Effect, % (95% CI, %: LL, UL)** | | |
| **Demographics and External Factors** | **Unwilling** | **Neutral** | **Willing** |
| Age, 1-YearIncrease | 0.0 (-0.4, 0.3) | 0.0 (-0.1, 0.1) | 0.0 (-0.4, 0.5) |
| Comorbidity Score, 1-Unit Increase | -0.9 (-5.2, 3.4) | -0.2 (-1.5, 1.0) | 1.1 (-4.4, 6.7) |
| Urban Residence (Ref: Rural) | -1.3 (-9.8, 7.3) | -0.3 (-2.7, 2.0) | 1.6 (-9.3, 12.5) |
| Race (Ref: White/Caucasian) |  |  |  |
| Black/African American | 2.2 (-13.7, 18.0) | 0.6 (-3.6, 4.8) | -2.8 (-22.8, 17.3) |
| Other | -8.4 (-20.9, 4.2) | -3.3 (-9.6, 2.9) | 11.7 (-6.9, 30.3) |
| Non-Response^a^ | 20.9 (-14.1, 55.9) | 2.4 (0.3, 4.6) | -23.3 (-57.3, 10.6) |
| Hispanic Ethnicity  (Ref: Not Hispanic) |  |  |  |
| Yes | -6.6 (-30.5, 17.3) | -2.3 (-12.4, 7.7) | 8.9 (-24.9, 42.8) |
| Non-Response^a^ | 13.2 (-44.3, 70.7) | 2.2 (-1.6, 5.9) | -15.3 (-76.3, 45.7) |
| Any Unmet Needs^b^ (Ref: None) | 14.0 (1.5, 26.6) | 2.9 (0.7, 5.2) | **-17.0 (-31.2, -2.7)** |
| Lack of Transportation Frequency (Ref: Never) |  |  |  |
| Often | -12.1 (-33.0, 8.8) | -4.4 (-15.0, 6.2) | 16.5 (-14.8, 47.8) |
| Sometimes | -10.9 (-21.7, -0.2) | -3.9 (-8.6, 0.8) | 14.8 (-0.4, 30.0) |
| Rarely | -3.8 (-18.3, 10.7) | -1.1 (-5.5, 3.4) | 4.9 (-14.1, 23.8) |
| **Perceived Ease of Use** |  |  |  |
| Internet Access By  (Ref: Broadband Internet) |  |  |  |
| Cell Phone Only | 20.6 (-6.6, 47.8) | 2.7 (0.9, 4.5) | -23.3 (-50.0, 3.4) |
| Satellite | 0.0 (-16.2, 16.2) | 0.0 (-5.1, 5.1) | 0.0 (-21.3, 21.2) |
| I have no Internet at home | 12.4 (-7.2, 32.1) | 2.4 (0.2, 4.7) | -14.9 (-36.5, 6.7) |
| Used a computer at home, at work, or at school. (Ref: No) | 14.2 (1.8, 26.6) | 6.0 (-1.2, 13.3) | -20.2 (-39.5, -0.9) |
| Has an unlimited data plan.  (Ref: No) |  |  |  |
| Yes | 0.9 (-12.2, 14.0) | 0.3 (-3.8, 4.3) | -1.1 (-18.3, 16.0) |
| I don’t have a cell phone. | 31.4 (-1.4, 61.3) | 1.6 (-4.8, 8.1) | **-33.0 (-60.3, -5.6)** |
| Non-Response^a^ | 23.2 (-16.4, 62.8) | 2.5 (-2.2, 7.2) | -25.7 (-64.4, 13.0) |
| **Perceived Usefulness** |  |  |  |
| Interested in telemedicine but don’t know how to use it. (Ref: No) |  |  |  |
| Yes | -15.0 (-24.8, -5.1) | -6.2 (-12.0, -0.3) | **21.1 (5.9, 36.4)** |
| Non-Response^a^ | -3.6 (-33.0, 25.8) | -1.0 (-10.4, 8.4) | 4.6 (-34.1, 43.4) |
| Used telemedicine before the COVID-19 pandemic. (Ref: No) | 2.3 (-6.3, 10.8) | 0.6 (-1.7, 3.0) | -2.9 (-13.8, 8.0) |
| Comfortable using the telemedicine technology. (Ref: No) |  |  |  |
| Yes | **-17.6 (-33.7, -1.5)** | **-3.0 (-4.9, -1.1)** | **20.6 (3.6, 37.6)** |
| No telemedicine visits in past year | -9.0 (-28.8, 107.0) | -0.9 (-3.2, 1.5) | 9.9 (-11.6, 31.4) |
| A telemedicine visit would be easier for me if I had: (Ref: No) |  |  |  |
| Help getting access to the Internet | 2.0 (-14.3, 18.3) | 0.5 (-3.7, 4.7) | -2.5 (-23.0, 17.9) |
| Help getting access to a tablet or other device | -10.1 (-23.4, 3.2) | -3.4 (-8.4, 1.7) | 13.5 (-4.8, 31.7) |
| Other | -11.0 (-22.3, 0.3) | -4.3 (-10.0, 1.3) | 15.3 (-1.5, 32.1) |
| Communicated with doctor via (Ref: Email or Video Telemedicine) |  |  |  |
| In-person or Via Telephone Only | 9.2 (-5.2, 23.6) | 2.1 (-0.6, 4.7) | -11.2 (-28.1, 5.6) |

a. Non-Response includes don’t know and refused responses. For variables with ≥5 non-response answers, a category of “non-response” was included; else non-response was treated as missing.
b. This includes at least one of the following: Food, Clothing, Utilities, Childcare, Medicine or Healthcare, Phone, or Other
c. Bolded differences are significant at the 0.05 level
